# A new species of genus *Chorebus* Haliday (Hymenoptera, Alysiinae) parasitising *Hexomyza caraganae* Gu (Diptera, Agromyzidae) from NW China

**DOI:** 10.3897/zookeys.663.11874

**Published:** 2017-03-28

**Authors:** Tao Li, Cornelis van Achterberg

**Affiliations:** 1 General Station of Forest Pest Management, State Forestry Administration, Shenyang 110034, P. R. China; 2 Shaanxi Key Laboratory for Animal Conservation / Key Laboratory of Resource Biology and Biotechnology in Western China, College of Life Sciences, Northwest University, 229 North Taibai Road, Xi’an, Shaanxi 710069, China; 3 Department of Terrestrial Zoology, Naturalis Biodiversity Center, Postbus 9517, 2300 RA Leiden, The Netherlands

**Keywords:** Alysiinae, biology, *Caragana korshinskii*, *Chorebus*, Dacnusini, *Hexomyza caraganae*, host, new species, parasitoid, twig gall

## Abstract

Chorebus (Stiphrocera) hexomyzae **sp. n.** (Hymenoptera, Braconidae, Alysiinae, Dacnusini) is described and illustrated. It was reared from twig galls of *Hexomyza caraganae* Gu (Diptera, Agromyzidae) on *Caragana korshinskii* Kom. f. (Fabaceae) in Ningxia and Inner Mongolia (NW China). A partial key to related or similar *Chorebus* species is provided.

## Introduction

The subfamily Alysiinae (Hymenoptera, Braconidae) is a large and common subfamily containing 2,440+ valid species worldwide (Yu et al. 2016). The subfamily is characterized by having mandibles with 3 or 4 more or less outwardly curved (“exodont”) teeth (Fig. 7; Shaw and Huddleston 1991; van Achterberg 1993; Belokobylskij and Kostromina 2011). Rarely, the mandibles have up to 5–7 teeth or lobes, or possess only 1–2 teeth; in all cases the mandibles, if they are closed, do not touch each other. Traditionally, the Alysiinae are divided into two tribes: Alysiini and Dacnusini. The tribe Alysiini contains 76 valid genera and nearly double the number of species compared to the Dacnusini with only 31 valid genera (Yu et al. 2016). The presence (Alysiini) or absence (Dacnusini) of vein r-m of the fore wing is the main morphological difference between the two tribes, with wingless or brachypterous specimens included in the Alysiini. In general, Alysiini are koinobiont endoparasitoids of larval cyclorrhaphous Diptera in moist substrates like dung, carcasses or other decaying organic matter (Wharton 1984; Shaw and Huddleston 1991). In contrast, Dacnusini are koinobiont endoparasitoids of larval cyclorrhaphous Diptera mining in leaves or stems (Yu et al. 2016). The new species of *Chorebus* Haliday, 1833, (Alysiinae, Dacnusini) belongs to a large cosmopolitan genus with 460 valid species (Yu et al. 2016), but most species are described from the northern hemisphere. The new species is peculiar because of its association with a dipterous twig-galler. To date, it is only the second known reliable host association of a *Chorebus* sp. with a twig-galler. Up to now, only *C. gedanensis* (Ratzeburg, 1852) was reared multiple times from the poplar twig gall fly, *Hexomyza schineri* (Giraud, 1861) in Europe (Nixon 1937, 1944; Griffiths 1967; Georgiev 2004). The Nearctic *Chorebus agromyzae* (Gahan, 1913) is recorded from the same host on willow, but this is based solely on a reference by Fulmek (1968) which is most likely incorrect considering the host association in the original description by Gahan (1913), viz., Cerodontha (Butomomyza) angulata (Loew, 1869), a species leaf mining *Carex* spp. The two references concerning galls of Cynipidae by Rudow (1918) are obviously erroneous as hymenopterous larvae are not parasitized by Alysiinae. For the recognition of the subfamily Alysiinae, see van Achterberg (1976, 1990, 1993) and for additional references see Yu et al. (2016).

## Materials and methods

Twig galls of *Caragana korshinskii* Kom. f. (Fabaceae) were collected in Ningxia and Inner Mongolia (NW China), and placed in a large nylon cage at room temperature in the laboratory. Distilled water was sprayed over the galls twice a week to prevent desiccation and the emerged insects were collected daily. The galls were induced by *Hexomyza caraganae* Gu (Diptera: Agromyzidae); the inducer was kindly identified by Dr. Guang-Chun Liu (Shenyang University, Shenyang).

For the morphological terminology used in this paper, see van Achterberg (1993) and Harris (1979). The descriptions, measurements and figures were made using a Leica M205A microscope with a Leica Microsystem DFC550 digital camera. Photographs were combined using Leica Application Suite (Version 4.5.0).

The holotype and some paratypes are deposited at the Department of Life Sciences, Northwest University, Xi’an (**NWUX**), China. Most paratypes and hosts are deposited in the Insect Museum of the General Station of Forest Pest Management (**GSFPM**), State Forestry Administration, Shenyang, China. Some paratypes are deposited at the Naturalis Biodiversity Center (**RMNH**), Leiden.

## Results

### Key to Palaearctic species reared from *Hexomyza* Enderlein and similar species

**Table:** 

1	First metasomal tergite 1.1–1.3 times as long as its apical width and its apical half distinctly widened posteriorly (Fig. 5; but subparallel-sided in *C. singularis*); hind coxa evenly setose dorsally (Figs 1, 6, 11); vein r of fore wing distinctly longer than width of pterostigma and strongly oblique (Fig. 2); vein 3-CU1 of fore wing 3.0 times longer than vein CU1b	**2**
–	First tergite 1.8–2.5 times as long as its apical width and its apical half nearly parallel-sided; basal half of hind coxa with dorsal tuft of dense setae; vein r of fore wing slightly shorter than width of pterostigma and moderately oblique; vein 3-CU1 of fore wing 1.5 times longer than vein CU1b; [marginal cell of fore wing slender, 3.0-3.5 times longer than its maximum width]	**5**
2	Two apical segments of maxillary palp nearly as long as height of head; hind coxa yellow; precoxal sulcus almost smooth; apical half of first metasomal tergite subparallel-sided; [mesoscutum punctulate and almost entirely setose]	***C. singularis* (Tobias, 1962)**
–	Two apical segments of maxillary palp 0.2–0.3 times as long as height of head; hind coxa black; precoxal sulcus at least distinctly narrowly crenulate; apical half of first tergite distinctly widened posteriorly (Fig. 5)	**3**
3	Palpi and hind femur dark brown; mandibles largely blackish; marginal cell of fore wing stout, 2.5 times longer than its maximum width (Fig. 2); mandible distinctly narrowed apically because of subbasally situated lower tooth (Figs 12–19); precoxal sulcus narrowly crenulate (Figs 1, 4); middle lobe of mesoscutum smooth, except some punctures (Fig. 4)	***C. hexomyzae* sp. n.**
–	Palpi and hind femur yellow; mandibles largely reddish brown; marginal cell of fore wing slender, 4–5 times longer than its maximum width; mandible subparallel-sided or slightly widened apically and lower tooth subapically situated; precoxal sulcus broadly crenulate; middle lobe of mesoscutum superficially rugose; [pronotum laterally and mesopleuron partly granulate]	**4**
4	Second–fourth segments of hind tarsus yellow, contrasting with its blackish telotarsus; lateral lobes of mesoscutum partly with short setae medially; first metasomal tergite without median carina	***C. coxator* (Thomson, 1895)**
–	Second–fourth segments of hind tarsus and its telotarsus dark brown; lateral lobes of mesoscutum glabrous except for some long setae laterally; first tergite usually with median carina	***C. nydia* (Nixon, 1937)**
5	Temple behind base of mandible rectangular, protruding, and nearly as wide as base of mandible; first metasomal tergite 2.2–2.5 times as long as its apical width	***C. gedanensis* (Ratzeburg, 1852)**
–	Temple behind base of mandible evenly curved and much narrower than base of mandible; first tergite 1.8–2.2 times as long as its apical width	**6**
6	Temple in dorsal view 1.4 times as long as eye; ovipositor sheath slightly projecting beyond apex of metasoma, its blackish part 0.7 times as long as hind basitarsus; occiput less densely setose	***C. ares* (Nixon, 1944)**
–	Temple in dorsal view nearly as long as eye; part of ovipositor sheath projecting beyond apex of metasoma approx. as long as second segment of hind tarsus, its blackish part 0.9 times as long as hind basitarsus; occiput more densely setose	***C. senilis* (Nees, 1812)**

## Taxonomy

### Chorebus (Stiphrocera) hexomyzae sp. n.

Taxon classificationAnimaliaORDOFAMILIA

http://zoobank.org/0025B40D-6DD5-4DB6-8897-51F5D4AC378E

Figures 1 , 2–11 , 12–19 , 20–21

#### Type material.

Holotype, ♀, (NWUX) “NW **China**: **Ningxia** Hui Autonomous Region, Shizuishan, Dawukou (N 39°06', E 106°20', 1140 m), 26.v.2015” and reared from *Hexomyza caraganae* Gu in twig galls on *Caragana korshinskii* Kom. f. Paratypes (88♀♀ 79♂♂): (GSFPM, NWUX, RMNH): 1♀, same data as holotype; 6♀♀ 3♂♂, id., but 17.v.2015; 5♀♀ 5♂♂, id., 23.v.2015; 3♀♀ 8♂♂, id., 24.v.2015; 4♀♀ 2♂♂, id., 25.v.2015; 5♀♀ 10♂♂, id., 27.v.2015; 1♀, id., 25.vi.2015; 3♀♀ 1♂, NW **China**: **Inner Mongolia** Autonomous Region, Hangjinqi (N 39°45', E 108°44', 1460 m), 22.v.2016; 4♀♀ 6♂♂, id., 23.v.2016; 1♂, id., 24.v.2016; 1♂, id., 26.v.2016; 2♀♀ 1♂, id., 27.v.2016; 1♀ 2♂♂, id., 28.v.2016; 2♀♀ 1♂, id., 30.v.2016; 2♀♀ 2♂♂, id., 31.v.2016; 1♀ 3♂♂, id., 2.vi.2016; 1♀, id., 3.vi.2016; 4♀♀ 4♂♂, id., 6.vi.2016; 1♀, id., 7.vi.2016; 1♀, id., 8.vi.2016; 2♂♂, id., 12.vi.2016; 15♀♀ 10♂♂, Inner Mongolia Autonomous Region, Dalate (N 40°17', E 109°54', 1020 m), 23–30.v.2016; 26♀♀ 17♂♂, id., 1–14.vi.2016.

**Figure 1.**
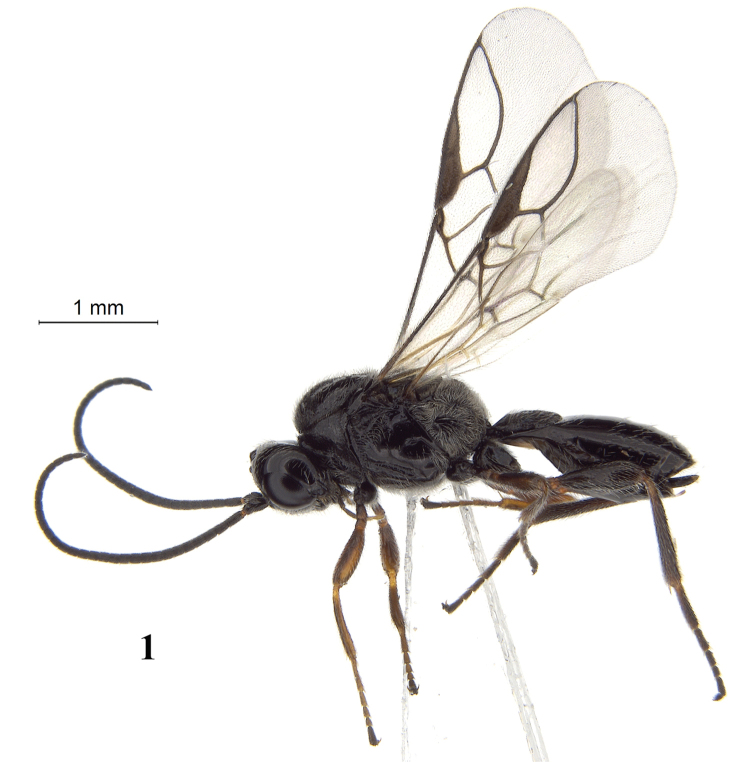
Chorebus (Stiphrocera) hexomyzae sp. n., female, paratype, habitus lateral.

#### Diagnosis.

**Figures 2–11.**
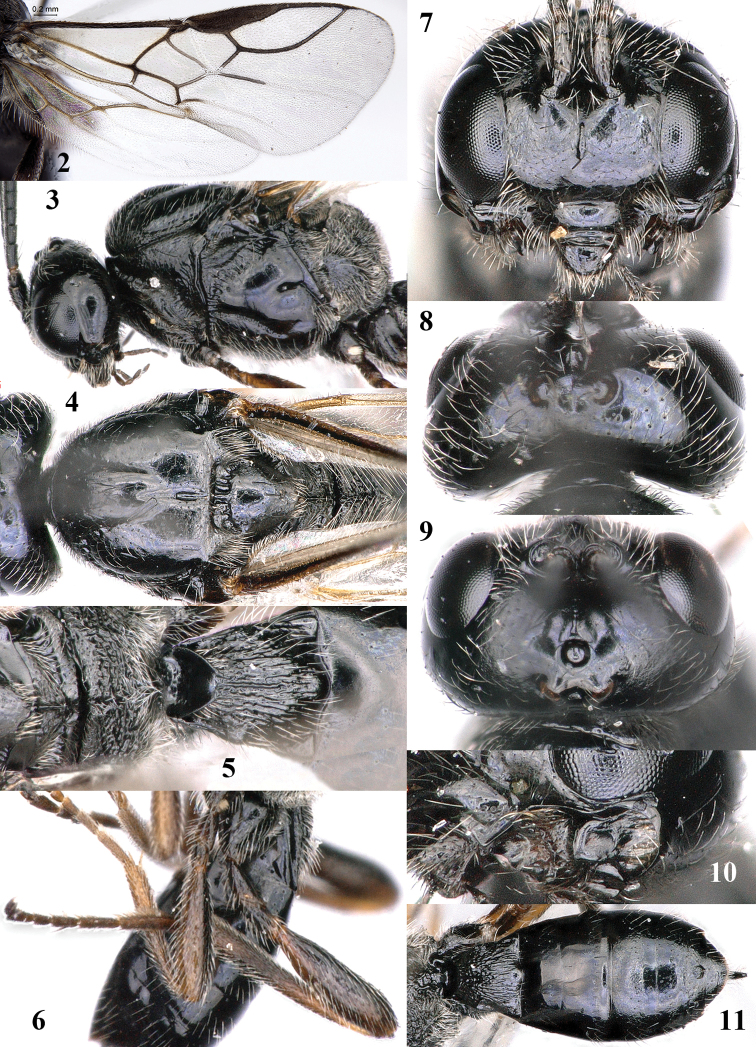
Chorebus (Stiphrocera) hexomyzae sp. n., female, holotype, but 2 of paratype. **2** Wings **3** Mesosoma lateral **4** Mesosoma dorsal **5** Propodeum and first metasomal tergite dorsal **6** Hind leg lateral **7** Head anterior **8** Head dorsal **9** Head antero-dorsal **10** Mandible, full view on middle tooth **11** Metasoma dorsal.

**Figures 12–19.**
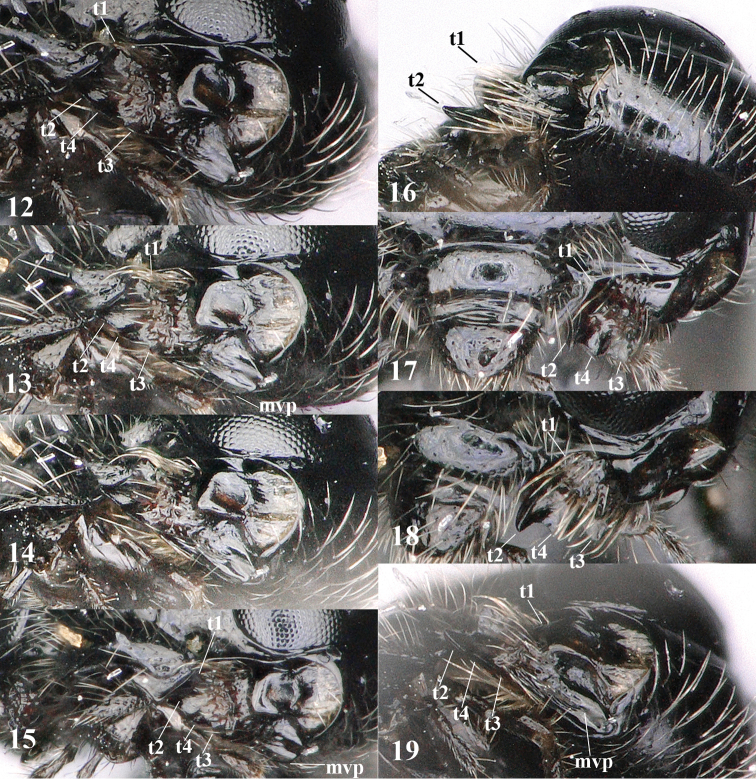
Chorebus (Stiphrocera) hexomyzae sp. n., female, holotype. **12–19**. Mandible at different angles; t1, t2, t3 = upper, middle and lower tooth, respectively; t4 = additional tooth on ventral side of middle tooth.

Antenna with 27–34 segments; eye in dorsal view 1.1–1.2 times as long as temple; temple medium-sized and rounded ventrally, moderately densely setose with medium-sized setae and hardly protruding behind base of mandible (Figs 4, 12, 14, 16, 19); third segment (including annellus) 1.4 times as long fourth segment; mandible with four teeth, middle tooth (= t2) wide triangular, acute, much longer than both lateral teeth, with an extra protuberance on ventral side of middle tooth and ventral (= t3) tooth rather close to base of mandible resulting in apically narrowed mandible (Figs 10, 12–19); notauli nearly complete and largely smooth (Fig. 4); lateral lobes of mesoscutum largely glabrous; length of vein r of fore wing almost equal to width of pterostigma (Fig. 2); vein CU1b short of fore wing short and first subdiscal cell closed and robust; vein 3-SR+SR1 rather short and regularly bent, resulting in a robust marginal cell (Fig. 2); first tergite slightly longer than its apical width, evenly convex and longitudinal rugae not obscured by setosity, dorsope small, and dorsal carinae united and connected with median carina (Fig. 5); second tergite smooth and posterior half sparsely setose (Fig. 11); setose part of ovipositor sheath 0.05 times as long as fore wing and 0.2 times as long as hind tibia (Fig. 1).

#### Description.

Holotype, ♀, length of body 3.9 mm, of fore wing 3.5 mm.

*Head.* Transverse and shiny in dorsal view, slightly widened posteriorly (Fig. 9), width of head 1.9 times its lateral length, in anterior view subcircular (Fig. 7), and 1.1 times wider than mesoscutum; antenna 0.7 times as long as fore wing and with 30 segments, short setose but apically with few long bristles, length of third segment (including annellus) 1.4 times as long as fourth segment, length of third, fourth and penultimate segments 2.6, 1.8 and 1.5 times their width, respectively (Figs 1, 3); length of maxillary palp 0.8 times height of head; eye in dorsal view 1.1 times as long as temple (Fig. 8); eye in lateral view 1.4 times higher than wide; frons convex laterally, remotely punctulate and setose, and slightly depressed behind antennal sockets and with shallow groove in front of anterior ocellus (Fig. 9); vertex rather convex and with long setae (Fig. 8); OOL:diameter of ocellus:POL= 14:7:8; face 1.4 times wider than high, rather evenly convex, with long setae and largely smooth, sparsely punctulate and with satin sheen; clypeus largely smooth, convex and transverse, depressed and slightly concave medio-ventrally (Fig. 7); malar space absent; mandible with four teeth, middle tooth (= t2) wide triangular, acute, much longer than both lateral teeth, with an extra protuberance on ventral side of middle tooth (similar to t3) and ventral (= t3) tooth rather close to base of mandible resulting in apically narrowed mandible (Figs 10, 12–19); medial length of mandible nearly equal its maximum width and mandible ventro-basally with large flat part nearly as wide as dorsal part of mandible (Figs 12–15, 19).

*Mesosoma.* Length of mesosoma 1.5 times its height; pronope wide, elliptical and large; side of pronotum largely smooth, sparsely setose and finely punctulate, only posterior half of oblique groove coarsely crenulate and some crenulae anteriorly (Fig. 3); mesoscutum without lateral carina in front of tegula, but with lateral groove (Fig. 4); tegula square and large; epicnemial area crenulate; precoxal sulcus narrow and finely crenulate, its posterior third absent (Fig. 3), remainder of mesopleuron smooth; pleural sulcus smooth; episternal scrobe medium-sized, oval and connected to pleural sulcus; metapleuron largely smooth dorsally and rugulose ventrally, without specialised central area, setae directing postero-ventrally, but setae of dorsal groove directing dorsally (Fig. 3); notauli nearly complete, smooth except some fine crenulae anteriorly and posteriorly separated from long and narrow linear medio-posterior depression (Fig. 4); lateral lobes of mesoscutum largely glabrous and with satin sheen, remainder of mesoscutum largely setose; scutellar sulcus deep and wide, with 3 long carinae and 2 short ones, sulcus 4 times wider than its median length; scutellum smooth and moderately convex, superficially impressed medio-posteriorly; axilla densely setose; metanotum with long setae and with regular and complete coarse median carina (Figs 4, 5); surface of propodeum rugose, medially with some coarse transverse rugae, with open setosity leaving sculpture well visible, but postero-laterally rather densely setose, anteriorly with short and rather weak median carina and areola absent (Fig. 5).

*Wings* (Fig. 2). Fore wing: r:2-SR:3-SR+SR1 = 10:14:49; 1-SR+M slightly sinuate; SR1 evenly bent (Fig. 2); r approx. equal width of pterostigma and oblique; cu-a postfurcal; 1-CU1:2-CU1 = 3:11; 3-CU1 much longer than short CU1b; m-cu antefurcal, nearly straight and distinctly converging to 1-M posteriorly; first subdiscal cell 2.1 times as long as wide; M+CU1 largely unsclerotised. Hind wing: M+CU:1-M:1r-m = 30:14:13; m-cu absent; cu-a straight.

*Legs.* Hind coxa largely smooth, without dense dorso-basal tuft of setae but with long whitish setae (Fig. 6); tarsal claws medium-sized, almost as long as arolium and with few bristles (Fig. 6); length of femur, tibia and basitarsus of hind leg 3.6, 9.0 and 4.4 times their width, respectively; length of hind tibial spurs 0.35 and 0.40 times their basitarsus; hind basitarsus slightly widened submedially (Fig. 6).

*Metasoma.* Length of first tergite 1.1 times its apical width, its dorsal carinae united at basal quarter and connected with median carina, medially evenly convex and rather regular and coarse longitudinal rugae distinctly visible despite long setosity (Fig. 5); dorsope small and round, laterope obsolescent; second tergite smooth and medio-anteriorly glabrous, remainder sparsely setose; setose part of ovipositor sheath 0.05 times as long as fore wing (total visible sheath 0.08 times), narrowed apically and 0.2 times as long as hind tibia (Fig. 1).

**Figures 20, 21.**
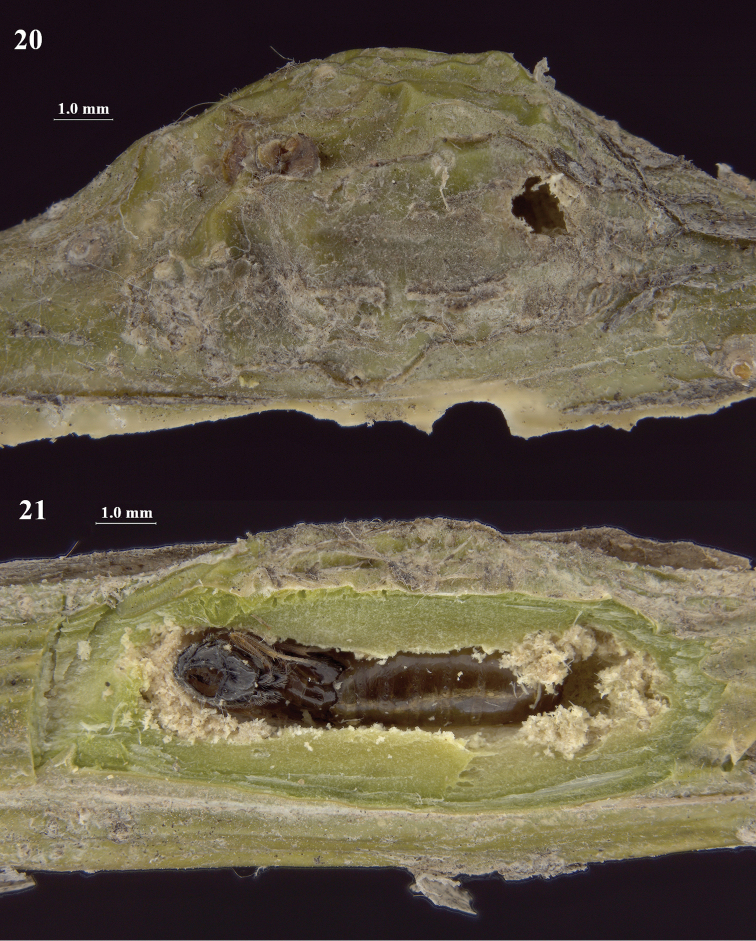
**20** Twig gall of *Hexomyza caraganae* Gu, with emergence hole of Chorebus (Stiphrocera) hexomyzae sp. n. **21** Adult of C. (S.) hexomyzae emerging from puparium of *H. caraganae* Gu.

*Colour.* Black (including mandible); palpi, legs (but coxa and dorsally femora black), pterostigma and veins dark brown; wing membrane subhyaline.

*Male.* Similar to female. Antenna with 3–4 segments more than in female and slightly slenderer.

#### Variations.

Length of body of ♀ 3.0–3.9 mm, and of fore wing 2.7–3.5 mm; length of body of ♂ 3.3–3.9 mm, and of fore wing 2.9–3.7 mm; antenna of ♀ with 25(1), 26(1), 27(5), 28(12), 29(19), 30(10), 31(7) segments; antenna of ♂ with 30(2), 31(6), 32(9), 33(16), 34(12), 35(1), 36(1) segments; first metasomal tergite 1.0–1.1 times longer than its apical width; setose part of ovipositor sheath 0.04–0.05 times as long as fore wing; setae of second tergite as subposterior row or also laterally present; lateral lobes of mesoscutum nearly completely glabrous or anterior third setose and remainder glabrous; femora and tibiae dark brown with blackish streaks or yellowish brown; palpi dark brown or yellowish brown; mandible black or dark brown.

#### Biology.

Larval endoparasitoid of *Hexomyza caraganae* Gu, 1991 (Diptera: Agromyzidae) in twig galls on *Caragana korshinskii* Kom. (Fabaceae).

#### Distribution.

Palaearctic China (Inner Mongolia, Ningxia).

#### Remarks.

The new species belongs to the subgenus Stiphrocera Foerster, 1863, because it has smooth hind coxa without a dorsal tuft, and runs in the key to Far East Russian species by Tobias (1998) to *Chorebus coxator* (Thomson, 1895) and *C. singularis* (Tobias, 1962). The new species is easily separated by its dark palpi, mandible and legs. In addition, the shape of the mandible and the mesosomal setosity are different as indicated in the key. The new species is very different from the only other named species reared from *Hexomyza* twig galls, the European *C. gedanensis* (Ratzeburg, 1852), because of the elongate first metasomal tergite and shorter vein r of the fore wing of the latter. Two similar species occurring in the East Palaearctic region (*C. ares* (Nixon, 1944) and *C. senilis* (Nees, 1812)) are included in the key for comparison.

#### Etymology.

The specific name is derived from the host’s generic name: *Hexomyza* Enderlein, 1936.

## Supplementary Material

XML Treatment for Chorebus (Stiphrocera) hexomyzae
